# Editorial: Natural products, medicinal foods, herbal medicines, bone metabolism diseases, bone-related tumor diseases

**DOI:** 10.3389/fphar.2024.1421115

**Published:** 2024-05-15

**Authors:** Jiyu Han, Xuekun Lv, Zitong Zhao, Yuting Liu, Daqian Wan

**Affiliations:** ^1^ Department of Orthopedics, Tongji Hospital, School of Medicine, Tongji University, Shanghai, China; ^2^ Key Laboratory of Spine and Spinal Cord Injury Repair and Regeneration, Ministry of Education, Shanghai, China; ^3^ Institute of International and Comparative Education, East China Normal University, Shanghai, China; ^4^ Department of Anesthesiology and Pain Rehabilitation, Shanghai YangZhi Rehabilitation Hospital (Shanghai Sunshine Rehabilitation Center), School of Medicine, Tongji University, Shanghai, China; ^5^ Department of Eighth Internal Medicine, Shenyang Traditional Chinese Medicine Hospital, Shenyang, China

**Keywords:** natural products, medicinal foods, complementary and alternative medicine, bone metabolism diseases, bone-related tumor diseases

The skeletal system is a crucial part of the human body. Bone Metabolism Diseases including osteoporosis, osteoarthritis, and osteoarthritis have become more and more prevalent and rampant throughout the world. The primary bone tumor and bone metastasis of tumor cells have gradually become a major threat to human health worldwide too. However, there are no effective means for diagnosing and treating bone metabolism and bone-related tumor diseases in modern medicine. To overcome this problem, Medicinal foods, Natural products, medicinal plants, and preparations derived from them are to be the focus of considerable research interest. Single metabolites derived from medicinal plants and foods are important. They have become an essential part of complementary and alternative medicine in recent years. It is well known, that their secondary metabolites are important for biological activities and pharmacological effects, such as the promotion of osteogenic and antitumor effects. Unfortunately, the pathogenesis of bone metabolism and bone-related tumor diseases have not been fully elucidated, making its pharmacological treatment difficult. Therefore, the study of disease pathogenesis and treatment mechanisms is very important.

When applying natural medicine in the traditional way to treat skeletal system diseases, natural medicine is usually a mixture. However, we find a diverse range of therapies using herbal medicines and herbal products and isolated metabolites derived from them. So, The use of natural medicines needs to be more precise and specific, involving the purification and isolation of single elements or compounds from natural medicines. Some compounds isolated from natural medicines have been reported as regulators of bone homeostasis in a multitarget manner. Cinobufagin, melittin, and baicalein exert antitumor activity and induce tumor cell apoptosis in osteosarcoma. Therefore, natural drugs require consideration for bone metabolism and bone-related tumor disease therapy. These natural remedies are particularly intriguing due to their rich content of bioactive metabolites, which play pivotal roles in osteogenic and antitumor activities. In this context, the exploration of medicinal foods, natural products, and derived medicinal plants has proliferated, providing new directions for the treatment of bone diseases.

In addition, the comprehensive study of natural products, focusing on their pharmacological evaluation, constituent analysis, clinical impacts, and mechanistic understanding, is crucial for advancing our capabilities in treating bone metabolism diseases and related tumor conditions effectively. This multidisciplinary approach not only broadens the therapeutic repertoire but also deepens our understanding of disease mechanisms, thereby facilitating the development of innovative and more natural treatment strategies.

A total of four articles were included in this Research Topic, including two original research articles, one systematic review article, and one review article.

Duan et al. investigate the core components, targets, and potential mechanisms of antibacterial properties and major chemical components of pharmacodynamic substances of Tea-Seed Oil (TSO) through utilized ultra-performance liquid chromatography-quadrupole time-of-flight mass spectrometry (UPLC-Q-TOF/MS), network analysis, and molecular docking. Remarkably, out of the 47 components analyzed, seven exhibited significant inhibitory effects on pathogens such as *E. coli*, *S. aureus*, *P. aeruginosa*, and C. albicans. The potential mechanisms underlying these antibacterial actions are believed to be associated with the PI3K-AKT, estrogen, MAPK, and IL-17 signaling pathways. The findings offer the potential to enhance our understanding of TSO’s utilization, provide a theoretical basis for developing antibacterial drugs and cosmetics derived from TSO, and lay a solid foundation for the clinical application of TSO products.

Chargo et al. research marks the first demonstration that Korean Red Ginseng (KRG) extract significantly impacts the gut-bone axis during oral glucocorticoid (GC) treatment in CD-1 mice. This extract not only prevents glucocorticoid-induced osteoporosis (GIO) as evidenced in a subcutaneous pellet model but also notably alters gut microbiota composition and moderately affects gut barrier function and immune cell populations. These findings imply that concurrent oral administration of KRG extract with oral GCs could be advantageous for bone health in humans, suggesting a novel therapeutic avenue for managing glucocorticoid-induced complications.

Kwon et al. Provided evidence for the combined therapeutic effect of herbal medicine (HM) and Western Medicine (WM) for osteoporosis in patients with rheumatoid arthritis (RA) in this systematic review. This study conducted a systematic review and meta-analysis of 15 randomized controlled trials. The data showed that compared to using WM (disease-modifying anti-rheumatic drugs or bisphosphonates) alone, administering additional HM resulted in higher bone mineral density (BMD) in RA patients with osteoporosis. This analysis suggests that HM could be a promising alternative treatment for mitigating bone loss in patients with rheumatoid arthritis (RA).

Sun et al. Offered an exhaustive summary of the botany, traditional applications, phytochemistry, pharmacology, and safety of Hypericum sampsonii Hance (H. sampsonii), a highly valued medicinal plant in China known for its diverse pharmacological effects. Pharmacological investigations reveal that H. sampsonii is effective in treating gastrointestinal and gynecological disorders, as well as traumatic injuries, corroborating its uses in traditional medicine. This efficacy is attributed to the presence of PPAPs, benzophenones, xanthones, and flavonoids within the plant. In summary, this review underscores the significant potential of H. sampsonii for therapeutic use and as a source for drug development, highlighting the need for continued research to validate and expand upon its medicinal properties.

This Research Topic respectively introduced the effective constituents and possible antimicrobial molecular mechanisms of TSO. The synergistic effect of KRG extract and GCs on the treatment of osteoporosis was verified by affecting the osteo-intestinal axis *in vivo*. Through meta-analysis, it was proved that the active ingredients of HM combined with anti-rheumatic drugs may have better efficacy in RA-induced bone loss. This review summarized the possible active compounds of Hypericum, which need further study to validate and expand upon its medicinal properties. Despite the limited number of manuscripts analyzed, four previous studies provided significant insights into natural products for treating bone-related diseases. The critical findings underlined the importance of the effective ingredients and molecular mechanisms of natural products. Future research should focus on investigating molecular mechanisms and the clinical translation of pharmacologically active components.

